# How a Melanism Mutant Responds to Different Temperatures: A Case Study in Ladybird Beetle *Harmonia axyridis* Demonstrating Thermal‐Induced Phenotypic Plasticity

**DOI:** 10.1002/ece3.72503

**Published:** 2025-11-14

**Authors:** Yuan‐Xing Sun, Chu‐Lin Zhang, Sen‐Shan Wang, Rui‐Yan Ma, Xian‐Wei Li, Ya‐Nan Hao, Wei‐Na Kong

**Affiliations:** ^1^ Biocontrol Engineering Laboratory of Crop Diseases and Pests of Gansu Province, College of Plant Protection Gansu Agricultural University Lanzhou Gansu China; ^2^ Shanxi Key Laboratory of Integrated Pest Management in Agriculture, College of Plant Protection Shanxi Agricultural University Taiyuan Shanxi China

**Keywords:** carotenoids, ladybird beetle, melanin level, spot occurrence frequency, thermal melanism

## Abstract

Many insect species demonstrate thermal‐induced phenotypic plasticity in melanin levels as an effective temperature adaptation strategy. *Harmonia axyridis* (Pallas) (f. *succinea* morph), known for its diverse elytra patterns, generally displays a reduction in the overall melanin level and spot number on both the dorsal cuticle of pupa and elytra as temperature increases. In our laboratory colony, we have discovered a novel melanism mutant (*ml*) that exhibits elevated melanin levels throughout most developmental stages compared to the wild type (*wt*). In this study, we examined whether *ml* displays similar thermal‐induced phenotypic plasticity as *wt* under different temperatures (17.5°C, 25°C, and 32.5°C). Our findings indicated that *ml*, inherited in a Mendelian autosomal recessive pattern, exhibited much smaller changes in the occurrence frequency of dark spots on dorsal surfaces of both the pupa and the elytra alongside temperature variations. Additionally, *ml* consistently had significantly higher melanin levels than *wt* across all three temperature conditions. Surprisingly, despite having larger dark spot areas on the elytra, *ml* had higher carotenoid concentrations than *wt* at 25°C and 32.5°C, while no significant difference was observed at 17.5°C. These results indicated that the melanism mutant *ml* of *H. axyridis* displays distinct thermal‐induced melanism plasticity compared to the wild type, which will enhance our understanding of the thermal responses of insects differing in genetic background.

## Graphical abstract

*ml*, inherited in a Mendelian autosomal recessive manner, is a novel melanism mutant of *Harmonia axyridis* . When compared to *wt*, *ml* exhibited vastly smaller changes in the occurrence frequencies of dark spots on both the dorsal cuticles of pupa and elytra in response to thermal variations. *ml* had very high melanin level even at 32.5°C and showed a higher elytra carotenoid level than *wt*.

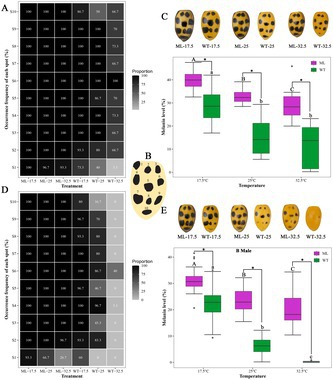

## 1 Introduction

Temperature is a pivotal abiotic factor that exerts significant influences on living systems (Angilletta Jr. 2009; Cossins and Bowler 1987; Vasudeva et al. 2019). Insects, being ectotherms, have evolved diverse strategies to regulate their body temperature (Heath et al. 1971; Heinrich 1993). An important non‐genetic strategy involves phenotypic plasticity achieved through alterations in cuticle pigmentation levels (Ma et al. 2021). Generally, when ambient temperature decreases, an increase in cuticular melanization is observed, which enhances thermal absorption (Rosa and Saastamoinen 2020; Solensky and Larkin 2003). Moreover, melanism is also the most prevalent form of phenotypic variation under normal rearing conditions (Liu et al. 2015; True 2003). Numerous spontaneously occurring melanism mutants have been observed in a wide range of insect species, with *Drosophila melanogaster* (True 2003) and *Bombyx mori* (Banno et al. 2005; Lu et al. 2003) being prominent examples. It is of great importance to explore whether the melanism mutants, characterized by genetic changes, display similar thermal‐induced phenotypic changes, and, meanwhile, exhibit higher levels of melanin across varying temperatures compared to the wild type. However, there is a paucity of research focusing on this aspect.

The multicolored Asian ladybird beetle, *Harmonia axyridis* (Coleoptera: Coccinellidae), native to Asia, has been widely used as a biocontrol agent (Koch 2003). However, it has also colonized several continents as an invasive species (Roy et al. 2016). *H. axyridis* is a paradigmatic example of an insect species that exhibits thermoregulatory phenotypic plasticity in pupal cuticle melanism. This phenotypic plasticity facilitated thermal adaptation has been suggested to be an important factor contributing to its global spread (Koch 2003; Camacho‐Cervantes et al. 2017). The f. *succinea* morph of *H. axyridis* , characterized by a yellow or red background with 0–19 dark spots on the elytra (Tan 1946), is the predominant phenotype in most colonized regions (Fiedler and Nedvěd 2019). The elytra pattern of *succinea* adults, resembling the dark spots observed on the dorsal cuticle of pupae, undergoes morphological changes in response to temperature fluctuations (Zhang et al. 2020). Within the temperature range of 14°C to 33°C, the levels of elytra melanization increase linearly with decreasing temperatures and decrease with increasing temperatures (Knapp and Nedvĕd 2013; Michie et al. 2011; Zhang et al. 2020). Specifically, the elytra melanination levels of male adults exhibit greater sensitivity to temperature changes (Knapp and Nedvĕd 2013; Zhang et al. 2020). In our laboratory colony of the f. *succinea* strain, we discovered several individuals exhibiting melanized cuticle coloration throughout most developmental stages. This melanism strain (named *ml*) appears to be a newly identified mutation, as evidenced by the hereditary pattern observed across multiple inbreeding generations. This mutant *ml* seems to be an ideal model to explore the thermal‐induced phenotypic plasticity of insect melanism mutants.

The elytra pattern of *H. axyridis* is formed through the deposition of melanin pigment (dark spots) and carotenoids (yellow‐orange to red background) (Bezzerides et al. 2007). Specifically, melanin synthesis occurs only in dorsal epidermal cells, while carotenoids accumulate only in ventral epidermal cells of the complementary regions (Ando et al. 2018). Therefore, it has been postulated that an unknown interaction between dorsal and ventral cells regulates the pigmentation processes involving melanin and carotenoids (Niimi and Ando 2021). In a previous study by Sun et al. (2018), they observed a significantly higher elytra carotenoid concentration in the light‐colored mutant *gr* compared to the wild type. Here, we questioned whether the melanism mutant *ml*, with an expanded dark spot area, exhibits a distinct carotenoid concentration compared to the wild type. Additionally, we explored whether both phenotypes show similar changes in carotenoids deposition in response to temperature variations.

In this study, a series of classical crossing experiments were first conducted to elucidate the inheritance pattern of the mutant *ml*. Subsequently, *ml* and *wt* larvae were reared under three different temperatures, and the occurrence frequencies of dark spots, along with the overall melanin level, on the dorsal surface of pupa and elytra were measured. Additionally, the carotenoid concentration in the elytra of newly emerged adults was measured. These results will provide novel perspectives for understanding how *H. axyridis* adapts to temperature changes based on phenotypic plasticity.

## 2 Materials and Methods

### 2.1 Insect Rearing and Strain Establishment

The multicolored Asian ladybird beetle, *H. axyridis* (f. *succinea* morph) was continuously reared with the pea aphid *Acyrthosiphon pisum* Harris within a controlled insectary (25°C ± 1°C, 65% RH and 14:10 h L: D). However, in October 2020, several aberrant third instar larvae exhibiting darkened cuticles (named *ml*) were discovered within the indoor‐reared population. These melanized larvae were collected and raised with *A. pisum* in a plastic Petri dish (9 cm in diameter). Newly emerged adults were paired, and the phenotype of their offspring was monitored. Notably, all larvae consistently manifested a melanism phenotype throughout all developmental stages (except for the first instar where it was less pronounced). Identical results were observed in subsequent generations following the inbreeding of *ml* individuals. These findings strongly suggest that *ml* is a novel heritable melanism mutant, and its population was successfully established with *A. pisum* in the insectary.

**FIGURE 1:**
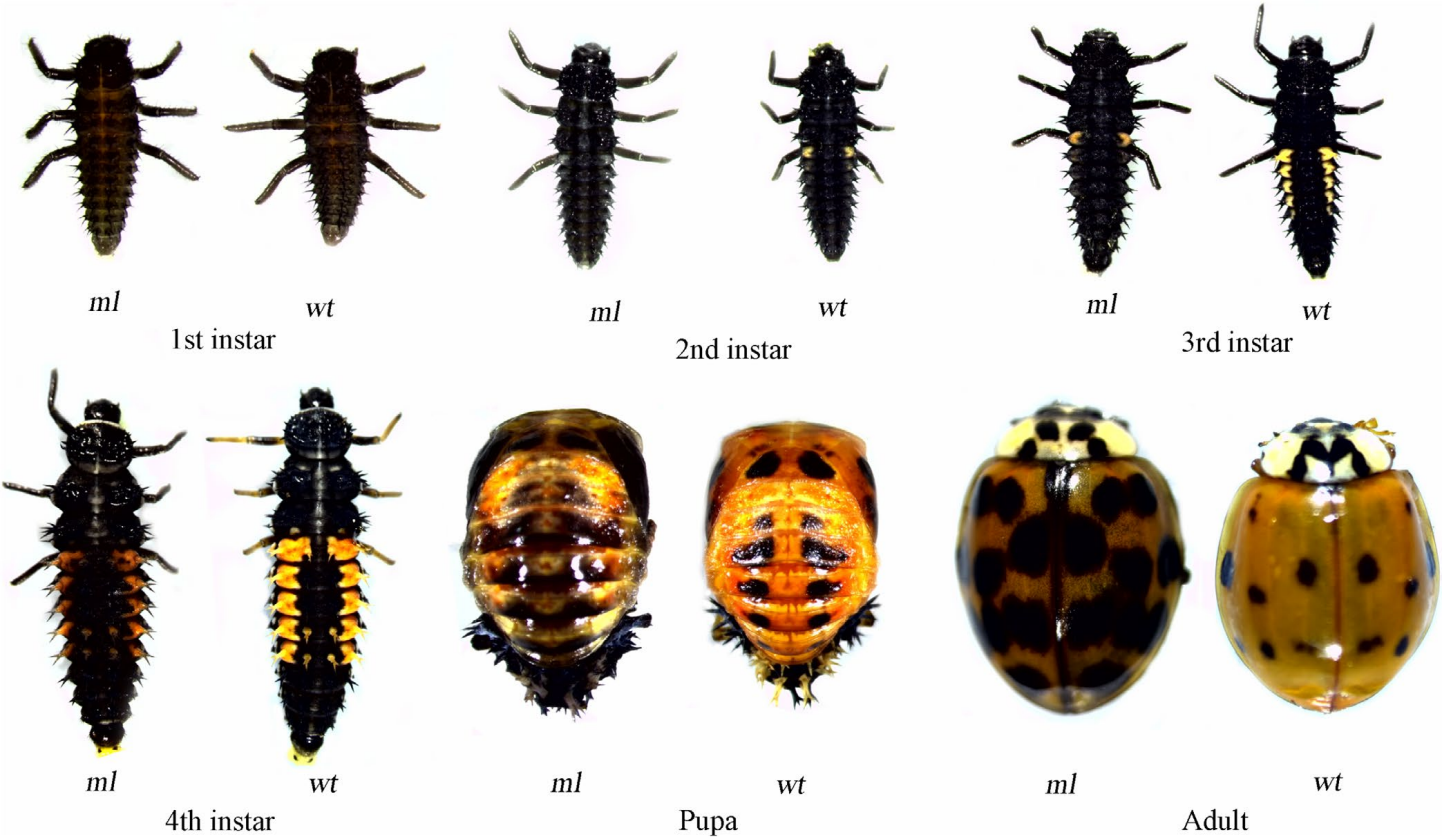
The typical morphology of different stages of *Hamonia axyridis* melanism mutant (*ml*) and wild type (*wt*). Photographs were taken from the dorsal view and under the same conditions, and the adults were female.

To establish a wild type (*wt*) population for conducting crossing experiments between the mutant *ml* and *wt*, in May 2021, 15 pupae were collected from an alfalfa field in Lanzhou, Gansu province (36°03′00″ N, 103°07′03″3E). After emergence, three pairs of newly emerged adults displaying f. *succinea* morph were continuously reared under the same conditions. The phenotypic differences between *ml* and *wt*, as visually observed, were illustrated in Figure 1 and were also reported in Li et al. (2022).

### 2.2 Crossing Experiments

The inheritance pattern of *ml* was determined following the methods described in Sun et al. (2018). Firstly, a series of single‐pair crosses between *ml* and *wt* were conducted to ascertain whether the mutant *ml* is a recessive allele. The couples were separately kept in clean plastic Petri dishes (9 cm in diameter) and supplied with sufficient *A. pisum* (approximately 100 mg). Additionally, a broad bean leaf was provided as an oviposition substrate. Daily inspections were carried out to monitor egg production. The eggs produced by each couple within 5 consecutive days were transferred to new Petri dishes (3 cm in diameter) for incubation. Newly hatched larvae were reared separately in obstructs‐equipped Petri dishes (9 cm in diameter) (Sun, Hao, Liu, et al. 2022; Sun, Hao, Zhou, et al. 2022), with six to nine individuals per dish. When the larvae reached the third instar stage, their phenotype was assessed based on cuticle coloration. Each cross involved eight pairs. Remarkably, we found that all heterozygous individuals displayed a normal coloration identical to that of the wild type, confirming that *ml* is a recessive mutant.

To further investigate whether the mutant *ml* is governed by a single locus or multiple loci, a series of single‐pair inbreeding crosses were conducted between the F1 hybrid offspring of *ml* and *wt*. Additionally, backcrosses were performed between the F1 heterozygotes and the presumed homozygous *mlml* individuals. The phenotype of their progeny was determined and recorded using the same methodology as described above.

### 2.3 Determination of the Development and Melanism Features Under Different Temperatures

Seventy newly hatched first instar larvae of *ml* as well as *wt* were individually reared in small plastic Petri dishes (3 cm in diameter), and they were subjected to 17.5°C, 25°C, and 32.5°C. For *ml*, the abbreviated names ML‐17.5, ML‐25, and ML‐32.5 were used to denote the treatments corresponding to these temperatures. Similarly, WT‐17.5, WT‐25, and WT‐32.5 were used for *wt*. In this study, 32.5°C was chosen as the high temperature regimen because temperatures close to 35°C are lethal to *H. axyridis*, as previously reported by Awad et al. (2021) and Knapp and Nedvĕd (2013). The rearing temperatures were achieved using three incubators (RDN‐300D‐5, Ningbo Southeast Instrument Co. LTD, Zhejiang, China) with 60% RH and a 14:10 h L:D photoperiod. During the feeding process, the larvae were provided with an adequate daily supply of *A. pisum* , with approximately 20 mg per first and second instar larva and 45 mg per third and fourth instar larva. Daily observation was conducted to monitor the survival rate and developmental stage of the beetle.

When reaching the pupa and adult stages, the melanin levels of the dorsal cuticle of pupae (24‐h old) and elytra (around 24 h after adult emergence) were measured following the methods described by Zhang et al. (2020). Specifically, digital camera images were captured from a top view of the pupae and the right elytra. Subsequently, the presence or absence of dark spots on each pupal segment (referred to Zhang et al. 2020) and each distribution site of the elytron (referred to by Michie et al. 2010) was recorded. Meanwhile, the proportions of dark spot area on the third thoracic segment (T3), the first to seventh abdominal segment (A1–A7), and the entire elytron were calculated using ImageJ (v. 1.8.0; Wayne Rasband; National Institute of Health, Bethesda, MD, United States). For each treatment, 30 images were utilized for analysis.

### 2.4 Measurement of Total Carotenoid Concentration in Elytra

The extraction and quantification of carotenoids in elytra was conducted according to the methods described by Bezzerides et al. (2007) and Li et al. (2023). Four randomly selected right elytra were weighed using a micro‐balance (Sartorius MSA 3.6P‐000‐DM, Gottingen, Germany) with an accuracy of 0.001 mg. Subsequently, the elytra were added to 1 mL of chloroform for carotenoid removal. After a 20‐min incubation on ice, the mixture was centrifuged at 13, 800 ***g*** for 5 min at 4°C. The supernatant was then carefully transferred into a new tube. After being dried under a nitrogen stream, the carotenoids were re‐dissolved in 500 μL alcohol (HPLC grade, Fisher A995‐4, ≥ 99.8%). The supernatant was measured at 450 nm using an absorbance reader (BioStack ready, USA). Carotenoid concentrations were determined based on a standard curve generated from serial dilutions of *β*‐carotene standard (> 98.0%) (CaroteNature, Switzerland). Each treatment had six replicates.

### 2.5 Statistical Analysis

All data analyses were conducted using R software 4.3.2 (R Core Team 2023). Analysis of the crossing experiments was conducted based on the null hypothesis that the melanism cuticle coloration is inherited in a simple Mendelian autosomal recessive manner. A chi‐squared test was run for each cross to determine whether the progeny ratio of *ml* to *wt* differed significantly from the expected ratio (1:3 for the inbreeding crosses of F1 hybrids and 1:1 for the backcross of F1 hybrids and recessive homozygotes). The survival rates under different temperatures were compared using a test of proportions (prop. test) (Sun, Hao, Liu, et al. 2022; Sun, Hao, Zhou, et al. 2022). Pairwise comparisons were performed using the pairwiseOrdinalIndependence function from the rcompanion package. For the melanin level and total elytra carotenoid concentration, non‐parametric Kruskal–Wallis tests were employed to assess differences among the three temperatures when data did not meet the assumptions of normality (Levene's test, *p* < 0.05). When a significant difference was detected, multiple comparisons were performed using the Mann–Whitney *U*‐test with Bonferroni correction (Ghazy et al. 2014). However, if the assumption of normality was satisfied, one‐way analysis of variance (ANOVA) was utilized, along with Tukey's HSD test for means separation (*p* < 0.05). At each temperature, the difference between *ml* and *wt* was analyzed with an independent‐sample *t*‐test (*p* < 0.05).

## 3 Results

### 3.1 Inheritance Pattern

Here, we hypothesized that the melanism mutant *ml* observed in our laboratory was inherited in a simple Mendelian autosomal recessive pattern. According to this hypothesis, the hybrid progeny of *ml* and the wild type (*mlml* × *wtwt*) would exhibit a wild phenotype with a frequency of 100%, and these heterozygotes should carry two alleles, one from each parent. Consequently, when the offspring siblings were mated, a melanism phenotype was predicted to occur in 25% of the F2 generation. Our results showed that mating between the F1 heterozygotes from *wtwt*♀ × *mlml*♂ (seven pairs) produced 118 and 368 offspring presenting melanism and normal phenotype, respectively. Similarly, mating between the F1 heterozygotes from *mlml*♀ × *wtwt*♂ (six pairs) produced 125 and 369 offspring with melanism and normal phenotype, respectively. These observed ratios did not significantly differ from the theoretical ratio of 1:3 (*χ* ^2^ = 0.134, *p* = 0.714; *χ* ^2^ = 0.024, *p* = 0.876) (Table 1). Additionally, when the F1 heterozygotes were crossed with the homozygous *mlml*, it was expected that 50% of the offspring would exhibit the melanism phenotype. Results from the backcross experiments (18 pairs in four types of combination) showed that the observed ratio of melanism (523) and normal offspring (535) aligned well with the theoretical ratio 1:1 (*χ* ^2^ = 0.136, *p* = 0.712) (Table 2). These results provided conclusive evidence that the mutation *ml* is homozygous recessive at a single locus.

**TABLE 1:** Frequencies of the melanism type (*ml*) and wild type (*wt*)offspring from the inbreeding crosses of the F1 heterozygotes of *wtwt* × *mlml*, with an expected ratio of 1:3 based on the null hypothesis that the mutant was formed with two recessive alleles. The exceptional significant *p* values were highlighted in bold.

Female parent	Male parent	No	Offspring phenotype		Expected morph ratio		Chi‐square test	
			#ML	#WT	#ML	#WT	*χ* ^2^	*p*
F1 *wtwt*♀ × *mlml*♂	F1 *wtwt*♀ × *mlml*♂	1	20	75	1	3	0.789	0.374
		2	**18**	**25**			**6.519**	**0.011**
		3	21	67			0.061	0.806
		4	17	55			0.074	0.785
		5	19	56			0.004	0.947
		6	8	45			2.774	0.096
		7	15	45			0	1.000
		Total	118	368	1	3	0.134	0.714
F1 *mlml*♀ × *wtwt*♂	F1 *mlml*♀ × *wtwt*♂	1	32	66	1	3	3.061	0.08
		2	24	84			0.444	0.505
		3	19	66			0.318	0.573
		4	13	57			1.543	0.214
		**5**	**18**	**29**			**4.433**	**0.035**
		6	19	67			0.388	0.534
		Total	125	369	1	3	0.024	0.876
In total			**243**	**737**	**1**	**3**	**0.022**	**0.883**

**TABLE 2:** Frequencies of wild type (*wt*) and melanism type (*ml*) offspring from the backcrosses of the F1 heterozygote of *wtwt* × *mlml* and homozygote *mlml*, with an expected ratio of 1:1 based on the null hypothesis that the mutant was formed with two recessive alleles.

Female parent	Male parent	No	Offspring phenotype		Expected morph ratio		Chi‐square test	
			#ML	#WT	#ML	#WT	*χ* ^2^	*p*
F1 *wtwt*♀ × *mlml*♂	P0 *mlml*	1	14	11	1	1	0.360	0.549
		2	31	30			0.016	0.898
		3	23	27			0.320	0.572
		4	64	57			0.405	0.525
		Total	132	125	1	1	0.191	0.662
P0 *mlml*	F1 *wtwt*♀ × *mlml*♂	1	3	4	1	1	0.143	0.70
		2	20	12			2.000	0.157
		3	32	46			2.513	0.113
		4	48	49			0.010	0.919
		5	51	66			1.923	0.166
		Total	154	177	1	1	1.598	0.206
F1 *mlml*♀ × *wtwt*♂	P0 *mlml*	1	12	14	1	1	0.154	0.695
		2	17	19			0.111	0.739
		3	29	33			0.258	0.611
		4	46	48			0.043	0.837
		5	46	31			2.922	0.087
		Total	150	145	1	1	0.085	0.771
P0 *mlml*	F1 *mlml*♀ × *wtwt*♂	1	15	17	1	1	0.125	0.724
		2	7	11			0.889	0.346
		3	46	48			0.043	0.837
		4	19	12			1.581	0.209
		Total	87	88	1	1	0.006	0.940
In total			**523**	**535**	**1**	**1**	**0.136**	**0.712**

### 3.2 Survival Under Different Temperatures

The pupal and adult survival rates of both *ml* and *wt* reared under 32.5°C were significantly lower than those of the same group reared under 17.5°C or 25°C (*ml‐*pupa: *χ* ^2^ = 18.52, *p* < 0.001; *ml‐*adult: *χ* ^2^ = 24.127, *p* < 0.001; *wt‐*pupa: *χ* ^2^ = 21.00, *p* < 0.001; *wt‐*adult: *χ* ^2^ = 22.24, *p* < 0.001) (Table 3). In terms of the comparison between *ml* and *wt*, their survival rates throughout all developmental stages did not exhibit significant differences regardless of the rearing temperatures (*p* > 0.05) (Table 3).

**TABLE 3:** Survival rates (%) of *Hamonia axyridis* melanism mutant (*ml*) and wild type (*wt*) under three different rearing temperatures.

Morph	Rearing temperature	Development stage					
		L1	L2	L3	L4	Pupa	Adult
*ml*	17.5°C	100 a	100 a	98.6 a	98.6 a	98.6 a	98.6 a
	25°C	100 a	100 a	100 a	100 a	98.6 a	95.7 a
	32.5°C	100 a	100 a	100 a	95.7 a	82.9 b	75.7 b
*wt*	17.5°C	100 A	100 A	100 A	100 A	100 A	97.1 A
	25°C	100 A	100 A	100 A	100 A	100 A	98.6 A
	32.5°C	100 A	100 A	100 A	100 A	85.7 B	78.6 B

*Note:* L1, L2, L3, and L4 represent the 1st, 2nd, 3rd, and 4th instar larva. Different uppercase and lowercase letters indicate significant differences between the three temperature treatments of *ml* and *wt*, respectively (*p* < 0.05).

### 3.3 Melanism Features Under Different Temperatures

#### 3.3.1 Pupa

**FIGURE 2:**
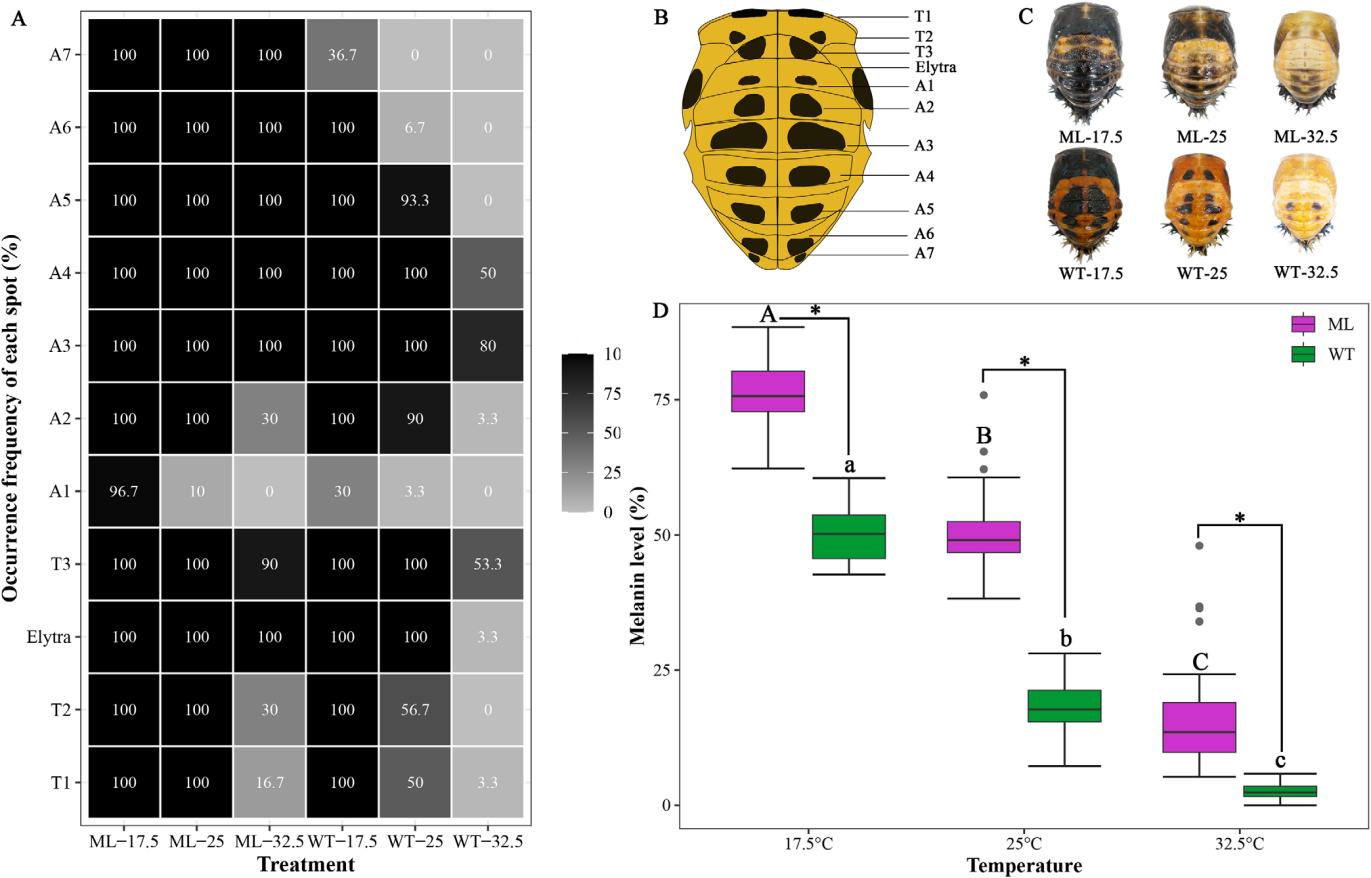
Thermal‐induced variation of the dark spots on the dorsal cuticle of the pupa of *Hamonia axyridis* melanism mutant (*ml*) and wild type (*wt*). The occurrence frequency of each spot (A), the distribution diagram of the dark spots (B), the typical phenotype of pupae (C, modified from Zhang et al. 2020), and the melanin level of the dorsal pupa (T3‐A7 segments). Different uppercase and lowercase letters indicate a significant difference between the three temperature treatments of *ml* and *wt*, respectively (*p* < 0.05). * indicates a significant difference between *ml* and *wt* at each rearing temperature (*p* < 0.05).

The distribution diagram of dark spots on the dorsal surface of the pupa was illustrated in Figure 2B. For *ml*, high temperature (32.5°C) induced a decrease in the occurrence frequency of dark spots on segments A1, A2, T1, and T2; however, dark spots stably occurred on all segments (except A1) under 17.5°C and 25°C (Figure 2A). Conversely, for *wt*, the occurrence frequencies of most dark spots decreased with increasing temperature. Specifically, when comparing 25°C with 17.5°C, there was a ≥ 50% decrease on segments A6 (from 100% to 6.7%) and T1 (from 100% to 50%); furthermore, when comparing 32.5°C with 25°C, a further ≥ 50% decrease was observed on segments A2 (from 90% to 3.3%), A4 (from 100% to 50%), A5 (from 93.3% to 0%), T2 (from 56.7% to 0%), and Elytra (from 100% to 3.3%) (Figure 2A). The typical phenotype of the pupae of *ml* and *wt* reared under different temperatures was shown in Figure 2C.

For both *wt* and *ml*, the melanin level of the dorsal segments of the pupa (T3‐A7) decreased significantly as the temperature increased (*wt*: Kruskal–Wallis *χ* ^2^ = 79.13, *p* < 0.001; *ml*: Kruskal–Wallis *χ* ^2^ = 76.34, *p* < 0.001). However, *ml* consistently exhibited a significantly higher melanin level than *wt* across the three rearing temperatures (17.5°C: *t* = 17.871, *p* < 0.001, 76.48% vs. 49.87%; 25°C: *t* = 18.833, *p* < 0.001, 51.29% vs. 17.44%; 32.5°C: *t* = 7.391, *p* < 0.001, 16.45% vs. 2.48%) (Figure 2D).

#### 3.3.2 Elytra of Adult

**FIGURE 3:**
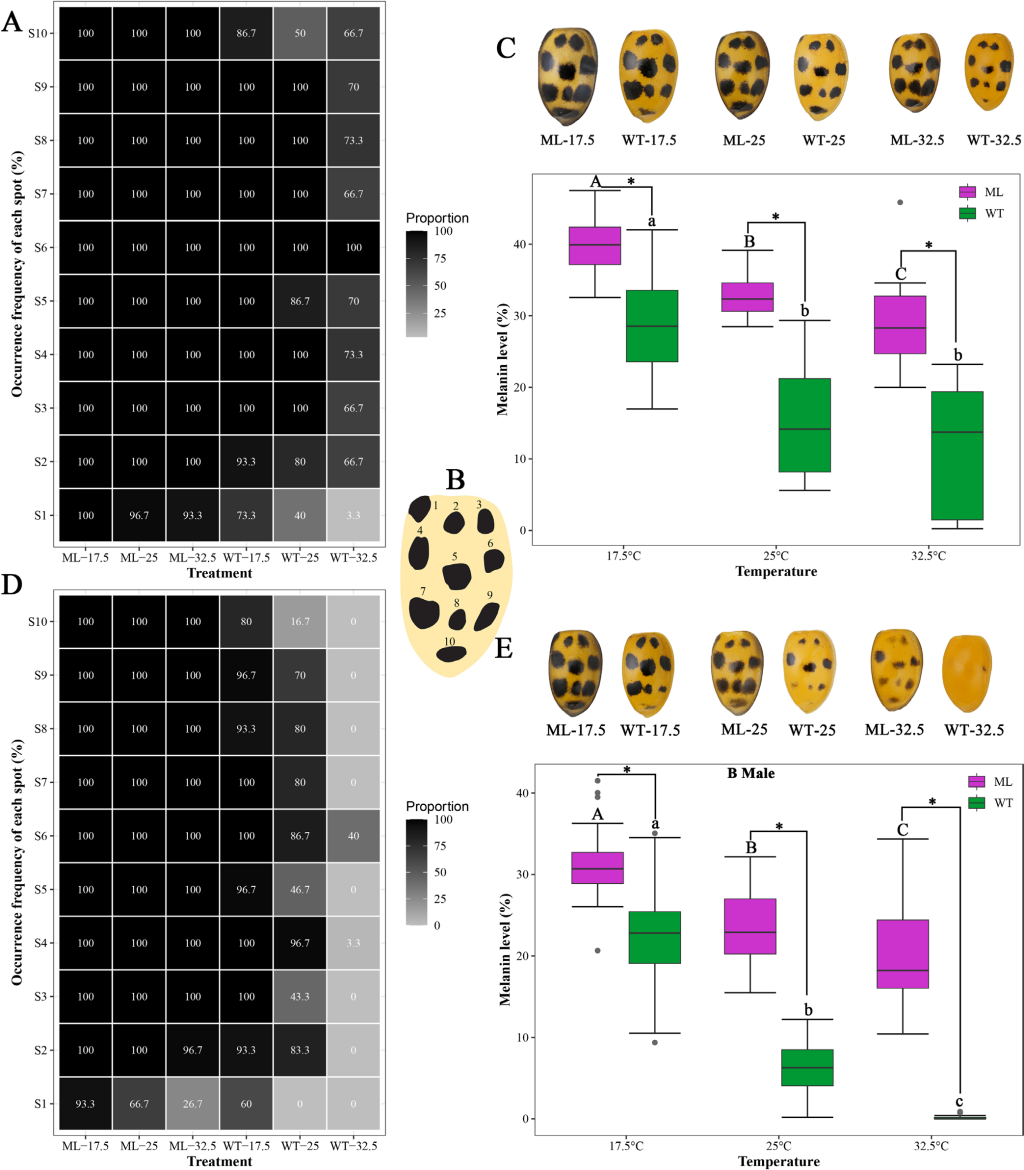
Thermal‐induced variation of the dark spots on elytra of *Hamonia axyridis* melanism mutant (*ml*) and wild type (*wt*). The occurrence frequency of each spot in female (A) and male (D) individuals, the distribution diagram of the dark spots (B, modified from Michie et al. 2010), the typical phenotype and melanin level of elytra of female (C) and male (E) individuals. Different uppercase and lowercase letters indicate a significant difference between the three temperature treatments of *ml* and *wt*, respectively (*p* < 0.05). * indicates a significant difference between *ml* and *wt* at each rearing temperature (*p* < 0.05).

The distribution diagram of dark spots on elytra was illustrated in Figure 3B. For *ml*, the occurrence frequencies of nearly all dark spots on the elytra of female *ml* individuals were 100% regardless of the rearing temperatures. The only exception was spot S1 in treatment ML‐25, which had an occurrence frequency of 96.7%, highly close to 100%. Conversely, for *wt*, when comparing 25°C with 17.5°C, there was a ≥ 30% decrease in the occurrence frequencies of spots S1 (from 73.3% to 40%) and S10 (from 86.7% to 50%); furthermore, when comparing 32.5°C with 25°C, there was a ≥ 30% decrease in the occurrence frequencies of spots S1 (from 40% to 3.3%), S3 (from 100% to 66.7%), S7 (from 100% to 66.7%), and S9 (from 100% to 70%) (Figure 3A). For both *ml* and *wt*, the melanin level of the elytra decreased as temperature increased. However, in *wt*, no significant difference was detected between the 25°C and 32.5°C treatments (*ml*: Kruskal–Wallis *χ* ^2^ = 51.278, *p* < 0.001; *wt*: *F*_2 87_ = 36.56, *p* < 0.001). Moreover, across the three rearing temperatures, *ml* consistently exhibited significantly higher melanin levels compared to *wt*, which can be visually observed from the typical elytra (17.5°C: *t* = 7.453, *p* < 0.001, 39.596% vs. 28.597%; 25°C: *t* = 37.067, *p* < 0.001, 32.973% vs. 15.400%; 32.5°C: *t* = 8.727, *p* < 0.001, 28.547% vs. 12.204%) (Figure 3C).

For males, most dark spots on the elytra of *ml* (except S1 and S2) maintained an occurrence frequency of 100% across all the three temperatures. Notably, the occurrence frequency of the spot S1 decreased significantly as temperature increased (from 93.3% at 17.5°C to 66.7% at 25°C, and then to 26.7% at 32.5°C). In contrast, the spot S2 still had a very high occurrence frequency of 96.7% at 32.5°C. However, for *wt*, the occurrence frequencies of all spots decreased as temperature increased. Specifically, almost all spots disappeared at 32.5°C (except for S4 with a low occurrence frequency of 3.3% and S6 with a moderate occurrence frequency of 40%). Additionally, spot S1 completely disappeared at 25°C (Figure 3D). The melanin level decreased with increasing temperature in both *ml* and *wt* groups (*wt*: Kruskal–Wallis *χ* ^2^ = 78.085, *p* < 0.001; *ml*: *F*_2 87_ = 40.09, *p* < 0.001). However, *ml* consistently exhibited significantly higher melanin levels than *wt* across the three temperatures, as evidenced by their typical elytra appearance (17.5°C: *t* = 6.081, *p* < 0.001, 31.292% vs. 22.764%; 25°C: *t* = 16.735, *p* < 0.001, 23.367% vs. 6.271%; 32.5°C: *t* = 17.97, *p* < 0.001, 16.94% vs. 0.15%) (Figure 3E).

### 3.4 Elytra Carotenoid Concentration

**FIGURE 4:**
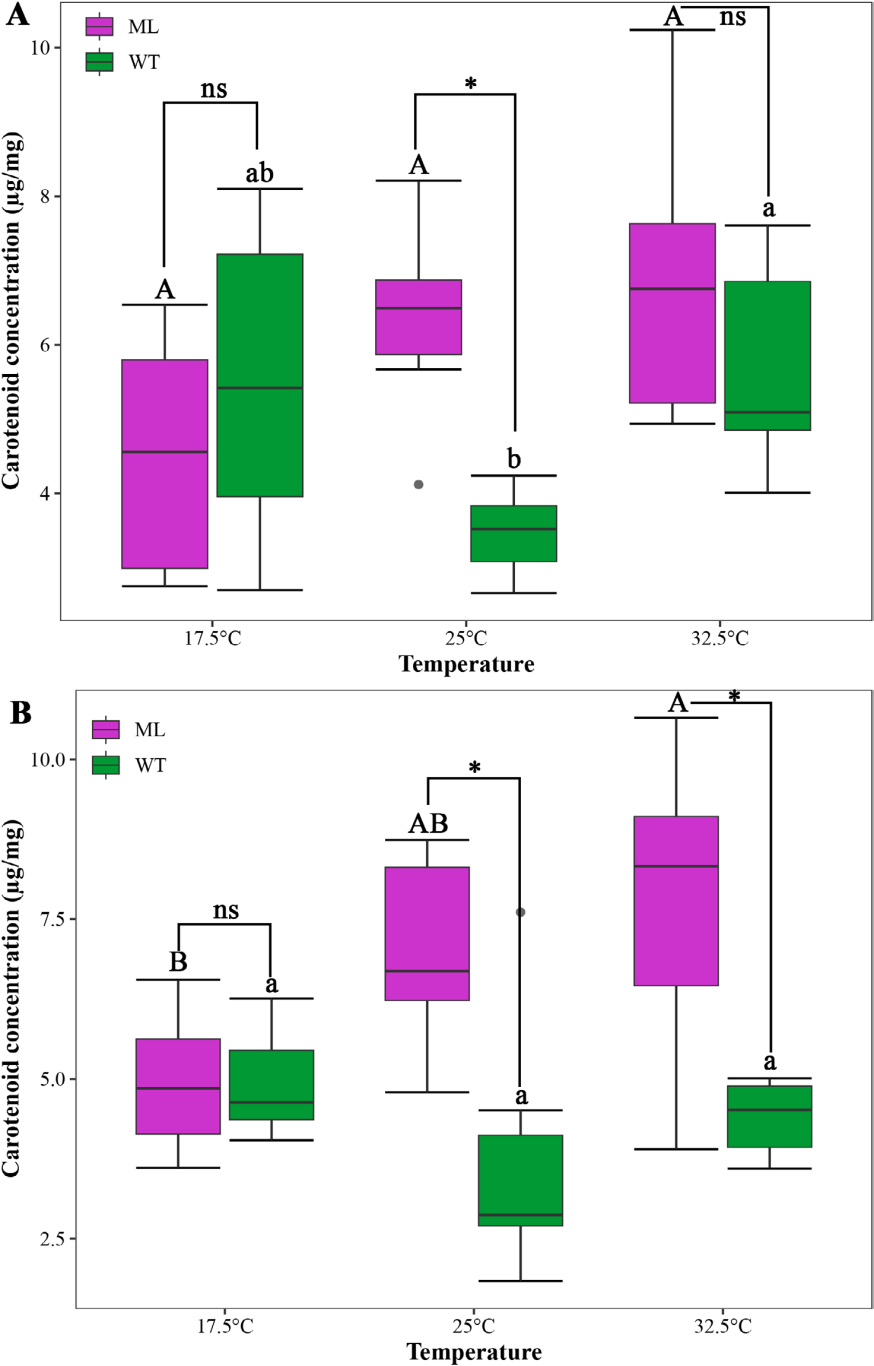
The elytra carotenoid concentration of female (A) and male (B) *Hamonia axyridis* melanism mutant (*ml*) and wild type (*wt*) under different rearing temperatures. Different uppercase and lowercase letters indicate a significant difference between the three temperature treatments of *ml* and *wt*, respectively (*p* < 0.05). * indicates a significant difference between *ml* and *wt* at each rearing temperature (*p* < 0.05), while ns means no significant difference (*p* > 0.05).

For *ml*, female individuals showed a slight increase in elytra carotenoid concentration as temperature increased, although no significant difference was observed (*F*_2,15_ = 3.137, *p* = 0.073). Similar trends were also detected in male individuals, and a significant difference was detected between 32.5°C and 17.5°C (*F*_2,15_ = 3.919, *p* = 0.043). However, for *wt*, individuals reared at 25°C showed a slight decrease in elytra carotenoid concentration compared to those reared at 32.5°C and 17.5°C. Moreover, a statistical difference between 25°C and 32.5°C was detected for female individuals (female: Kruskal–Wallis *χ* ^2^ = 6.589, *p* = 0.037; male: Kruskal–Wallis *χ* ^2^ = 3.521, *p* = 0.172) (Figure 4A,B).

When made a comparison between *ml* and *wt*, both female and male *ml* displayed a significantly higher elytra carotenoid concentration than *wt* under 25°C (female: *t* = 4.708, *p* = 0.002; male: *t* = 3.053, *p* = 0.013). Additionally, male *ml* also had a significantly higher elytra carotenoid concentration under 32.5°C (*t* = 3.262, *p* = 0.019) (Figure 4B) However, no statistical difference was detected between *ml* and *wt* under 17.5°C (female: *t* = −0.872, *p* = 0.405; male: *t* = 0.026, *p* = 0.980) (Figure 4).

## 4 Discussion

In this study, we have confirmed that *ml*, a novel melanism mutant in *H. axyridis* , follows a simple Mendelian autosomal recessive inheritance pattern. Unlike numerous insect melanism mutants that exhibit an abnormal phenotype at one or two developmental stages (Liu et al. 2015), *ml* displays a melanized cuticle coloration throughout all larval, pupal, and adult stages. Additionally, Sun et al. (2018) have reported a light‐colored mutant *gr*, which also exhibits abnormal coloration throughout all developmental stages. These findings suggest that the regulation of body color changes in mutants of *H. axyridis* , a ladybird beetle known for its elytra pattern polymorphism (Koch 2003; Komai et al. 1950; Komai 1956), might involve substantially different mechanisms compared to other insects and warrant further investigation.

Under three different rearing temperatures (17.5°C, 25°C, and 32.5°C), the occurrence frequencies of dark spots on the dorsal cuticle of the pupa (A2, A4, A5, T2, Elytra, A6, and T1) and elytra (S1, S3, S7, S9, and S10) of the wild type (*wt*) decreased as the temperature increased, which was highly in accordance with the changes observed under 15°C, 20°C, 25°C, and 30°C (Zhang et al. 2020). However, the mutant *ml* did not show a significant alteration in the occurrence frequency of dark spots across different temperatures. Specifically, even under the high‐temperature condition (32.5°C), only a partial disappearance of a few spots was observed, including those on A1, A2, T1, and T2 segments of the pupa and spot S1 of the elytra. These results strongly suggested that genetic changes in *ml* resulted in the thermal insensitivity of dark spot disappearance. Actually, Michie et al. (2010) have documented a genetic background‐based variation in the thermal reaction of *H. axyridis* . They found that melanic morphs show much smaller plasticity in elytra pattern compared to the non‐melanic morph (*succinea*). In the *succinea* morph, the dopamine pathway genes have been demonstrated to play crucial roles in melanin synthesis for both pupae and elytra, whereas the aspartate‐β‐alanine‐NBAD pathway genes have been verified to solely involve pupal melanization (Xiao et al. 2020; Zhang et al. 2020, 2021). Specifically, knocking down genes in the dopamine pathway generally leads to a complete disappearance of dark spots. In contrast, knocking down genes in the aspartate‐β‐alanine‐NBAD pathway (*ebony* and *ADC*) greatly enlarges the size of all spots on the dorsal cuticle of the pupa, even at the high temperature of 32.5°C (Xiao et al. 2020; Zhang et al. 2020, 2021). Nevertheless, the precise regulatory mechanisms underlying the thermal‐induced occurrence or disappearance of dark spots, particularly on elytra, remain largely unexplored. A comparative study between the wild type and the mutant *ml* would be invaluable for elucidating the molecular mechanisms.

Furthermore, our findings indicated that although *ml* demonstrated a decrease in melanin level of both the dorsal cuticles of pupa and elytra as temperature increased, similar to *wt*, a significantly higher value was detected across all rearing temperatures. Notably, *ml* reared at higher temperatures even displayed higher levels of melanin than *wt* reared at lower temperatures. For example, the melanin level of the dorsal cuticle of pupa was 17.44% for ML‐32.5, while it was 16.45% for WT‐25; similarly, a melanin level of 51.29% was detected for ML‐25, while it was 49.87% for WT‐17.5. The Thermal Melanism Hypothesis (TMH) proposes that body color darkness generally affects species' thermoregulatory performance by increasing heat absorption (Stuart‐Fox et al. 2017). As a result, darker individuals are predicted to be able to occur in colder environments than their lighter‐colored counterparts (Clusella‐Trullas et al. 2007). Here, both *ml* and *wt* demonstrated significantly reduced survival at 32.5°C compared to those at 17.5°C and 25°C. This finding confirms the detrimental impact of extreme high temperature on *H. axyridis* , as previously reported by Ma et al. (2021). However, it was worth noting that, under the 32.5°C regimen, the *ml* individuals with a higher melanin level did not demonstrate significantly different survival rates compared to the *wt* individuals. Above results suggested that the TMH effects on *H. axyridis* may be influenced or modulated by genetic background, as demonstrated in *Eoanthidium insulare* (Kasparek et al. 2024) and *Drosophila melanogaster* (Freoa et al. 2023). However, the melanic proportion of another ladybird beetle, *Adalia bipunctata* , has been found to be not only negatively correlated with temperature but, more importantly, with the duration of sunshine (Sloggett and Honěk 2012). Thus, a long‐term field observation should be conducted to reveal the melanism plasticity of *ml* under field conditions.

Surprisingly, *ml* demonstrated an increased concentration of elytra carotenoids along with a thermal‐induced reduction in melanin levels, while such a trend was not detected in *wt* individuals. Moreover, under 25°C conditions, *ml* displayed a higher elytra melanin level compared to *wt*, yet it had a significantly higher carotenoid concentration. However, when both *ml* and *wt* displayed very high melanin levels under a 17.5°C regimen, their elytra carotenoid concentrations were similar. Previous studies have proposed an intercellular interaction between elytra melanin pigmentation (in dorsal cells) and carotenoid pigmentation (in ventral cells) during pupal metamorphosis development (Ando et al. 2018; Ando and Niimi 2019). Our results suggested that the deposition of carotenoids in the elytra of *H. axyridis* may be influenced by its genetic background, thermal‐induced variation in melanization, and their interactions. It is important to note that most animals must acquire carotenoids through their diet (Koch and Hill 2018), and subsequently transport them to target tissues (Reboul and Borel 2011). Moreover, aside from their pigment function, carotenoids also perform crucial physiological roles. For example, they are important antioxidants (Smilanich et al. 2011; Wong and Svensson 2011). Consequently, the concentration of elytra carotenoids becomes a significant indicator of environmental adaptation. For example, reproductive females under long‐day conditions (14 L:10D) displayed a significantly higher elytra carotenoid concentration compared to diapause individuals subjected to short‐day conditions (10 L:14D) (Li et al. 2023). Therefore, regarding carotenoid deposition on the elytra, we can infer that *ml* has a superior environmental adaptation capability than *wt*.

In conclusion, our study demonstrated a spontaneously occurring melanism mutant in *H. axyridis* . This mutant displays different thermal‐induced phenotypic plasticity in dark spots on the dorsal cuticle of the pupa and elytra when compared to the wild type. These findings will enhance our understanding of the plasticity of melanism and its resultant effects in insects.

## Author Contributions

**Yuan‐Xing Sun:** methodology (equal), supervision (equal), writing – review and editing (equal). **Chu‐Lin Zhang:** investigation (equal). **Sen‐Shan Wang:** supervision (equal). **Rui‐Yan Ma:** supervision (equal). **Xian‐Wei Li:** data curation (equal), software (equal). **Ya‐Nan Hao:** writing – original draft (equal). **Wei‐Na Kong:** formal analysis (equal), software (equal).

## Conflicts of Interest

The authors declare no conflicts of interest.

## Data Availability

Data available from the Figshare Data Repository: https://doi.org/10.6084/m9.figshare.25727670.
